# Genome-wide analysis revealed the dysregulation of RNA binding protein-correlated alternative splicing events in myocardial ischemia reperfusion injury

**DOI:** 10.1186/s12920-023-01706-5

**Published:** 2023-10-19

**Authors:** Ning Ma, Hao Xu, Weihua Zhang, Xiaoke Sun, Ruiming Guo, Donghai Liu, Liang Zhang, Yang Liu, Jian Zhang, Chenhui Qiao, Dong Chen, Ailing Luo, Jingyun Bai

**Affiliations:** 1 Department of Cardiovascular Surgery, The First Affiliated Hospital of Zhengzhou University, Zhengzhou, 450052 Henan P.R. China; 2 Wuhan Ruixing Biotechnology Co., Ltd, Wuhan, 430206 Hubei P.R. China; 3 Department of Nephrology, The First Affiliated Hospital of Zhengzhou University, Zhengzhou, 450052 Henan P.R. China

**Keywords:** MIRI, RNA-seq, Alternative splicing, RNA binding protein, Correlation network

## Abstract

**Background:**

Myocardial ischemia reperfusion injury (MIRI), the tissue damage which is caused by the returning of blood supply to tissue after a period of ischemia, greatly reduces the therapeutic effect of treatment of myocardial infarction. But the underlying functional mechanisms of MIRI are still unclear.

**Methods:**

We constructed mouse models of MIRI, extracted injured and healthy myocardial tissues, and performed transcriptome sequencing experiments (RNA-seq) to systematically investigate the dysregulated transcriptome of MIRI, especially the alternative splicing (AS) regulation and RNA binding proteins (RBPs). Selected RBPs and MIRI-associated AS events were then validated by RT-qPCR experiments.

**Results:**

The differentially expressed gene (DEG) analyses indicated that transcriptome profiles were changed by MIRI and that DEGs’ enriched functions were consistent with MIRI’s dysregulated pathways. Furthermore, the AS profile was synergistically regulated and showed clear differences between the mouse model and the healthy samples. The exon skipping events significantly increased in MIRI model samples, while the opposite cassette exon events significantly decreased. According to the functional analysis, regulated alternative splicing genes (RASGs) were enriched in protein transport, cell division /cell cycle, RNA splicing, and endocytosis pathways, which were associated with the development of MIRI. Meanwhile, 493 differentially expressed RBPs (DE RBPs) were detected, most of which were correlated with the changed ratios of AS events. In addition, nine DE RBP genes were validated, including *Eif5*, *Pdia6*, *Tagln2*, *Vasp*, *Zfp36l2*, *Grsf1*, *Idh2*, *Ndrg2*, and *Uqcrc1*. These nine DE RBPs were correlated with RASGs enriched in translation process, cell growth and division, and endocytosis pathways, highly consistent with the functions of all RASGs. Finally, we validated the AS ratio changes of five regulated alternative splicing events (RASEs) derived from important regulatory genes, including *Mtmr3*, *Cdc42*, *Cd47*, *Fbln2, Vegfa*, and *Fhl2*.

**Conclusion:**

Our study emphasized the critical roles of the dysregulated AS profiles in MIRI development, investigated the potential functions of MIRI-associated RASGs, and identified regulatory RBPs involved in AS regulation. We propose that the identified RASEs and RBPs could serve as important regulators and potential therapeutic targets in MIRI treatment in the future.

**Supplementary Information:**

The online version contains supplementary material available at 10.1186/s12920-023-01706-5.

## Background

Acute myocardial infarction (AMI) is an acute ischemic heart disease caused by sudden occlusion of the coronary artery, resulting in myocardial ischemia, injury, and necrosis [1]. AMI is considered as a major fatal and disabling cardiovascular diseases (CVDs). Reperfusion is the most important treatment for myocardial infarction (MI), which can significantly reduce ischemic injury caused by AMI, limit the MI size, and lower the related mortality. However, reperfusion therapy can lead to myocardial ischemia-reperfusion injury (MIRI) while restoring the blood supply to the myocardium, reducing the efficacy of the treatment [2]. Thus, how to ameliorate MIRI has become a highly debated topic in CVD research [3]. The pathophysiological processes involved in MIRI are highly complex, including inflammation, endothelial dysfunction, oxidative stress, intracellular calcium overload, and mitochondrial dysfunction [2].

RNA binding proteins (RBPs) are a large group of proteins that can bind double- or single-stranded RNA and form ribonucleoprotein (RNP) complexes within cells. RBPs primarily function by binding with RNA and are often associated with RNA transcription initiation. At different stages of its life span, RNA binds to various types of RBPs to form RNP complexes, which regulate their maturation, transport, localization, and translation processes. Thus, diverse RBPs enable pivotal regulatory functions in almost all physiological and pathological processes [4]. Specifically, RBPs play vital roles in myocardial ischemia–reperfusion injury. For instance, QKI is an anti-apoptotic RBP in cardiac myocytes that facilitates survival under conditions of myocardial ischemia–reperfusion injury by antagonizing the elevation of specific pro-apoptotic factors [5]. Moreover, HMGB1, another RBP, plays a role in myocardial ischemia–reperfusion injury by triggering cardiomyocyte apoptosis through the TLR4 axis, promoting the release of cytokines and mediation of the inflammatory response via HMGB1/TLR4-related pathways [6]. Therefore, the present project aims to explore myocardial ischemia–reperfusion injury from the perspective of RBPs and offer novel insights for the diagnosis and treatment of this condition.

Many RBPs can bind pre-mRNA to regulate alternative splicing (AS) and produce proteins with various functions. AS refers to a process in which the 5′ and 3′ splice sites on the pre-mRNA of a transcribed gene are spliced together in different ways to generate multiple mRNA isoforms, resulting in protein products with different amino acid sequences that serve diverse physiological functions [7]. RBP-mediated AS regulation allows for the selection of different splicing sites, thus allowing a gene to produce multiple mRNAs and proteins to enhance the manner in which proteins can interact with one another [8, 9]. Aberrant AS is very common and plays an important role in the development of many human diseases [5]. Specifically, AS plays crucial roles in myocardial [10] and cerebral [11] ischemia–reperfusion injury. AS can add complexity to gene expression patterns, increase transcriptional efficiency, and promote protein diversity, thus playing an important role in various diseases. The RBP-AS regulatory network and its possible functions in MIRI have not yet been investigated with regard to a genome-wide landscape of RBPs.

Therefore, we proposed the scientific hypothesis that differentially expressed RBPs are involved in the post-transcriptional regulation processes after MIRI, which in turn causes variable splicing of pre-mRNAs. We hypothesised that the RBP-AS regulatory axis plays a molecular regulatory role in the MIRI process. In this study, we performed a systematic analysis of RNA-seq in mice with MIRI to obtain differentially expressed RBPs and variable splicing pre-mRNAs; we also combined with co-expression analysis to explore the genome-wide landscape of the RBP-AS regulatory network. The present study reveals a global picture of gene expression and splicing profiles as well as functional pathways in mouse models of MIRI. At present, there are a small number of reports on the role of RASEs and RBPs in the development of MIRI. In this view, we will discuss RBPs and its regulation of AS, and provide new mechanism insights for the development in MIRI.

## Methods

### MIRI model construction

The 6-week male C57BL/6 mice were originated from Hunan Slak Jingda. The mice were raised in the absence of specific pathogen free (SPF). The temperature of the feeding environment is 22 ~ 26 °C, the relative humidity is 50% ~ 60%, and the artificial light is dimmed for 12 h. For experiments, the mice were anesthetized by intraperitoneal injection of 0.12ml/10 g 1% sodium amytal. The hair on neck and chest was removed and the surgery site was disinfected. After oral tracheal intubation, the mice were connected to the ventilators to maintain respiration. The peak airway pressure was 14.40 cm H_2_O, the respiratory rate 88 times/min, and the ventilation volume 1 L/min. The skin of the surgical area was disinfected and cut off on the left side of the sternum between the third and fourth ribs. The subcutaneous tissue and muscles were passively separated layer by layer. The thoracic cavity was opened. The intercostals were opened by the chest opener to fully expose the heart. The pericardial sac was cut open by carefully identifying it under a stereomicroscope. A pink blood vessel appeared at the lower edge or left side of the auricle, which is the anterior descending branch (LAD) of the left coronary artery. Use a needle to pass a 6 − 0 suture through the surface layer of myocardium at 1 mm from the lower edge of the auricle and ligate the LAD under pressure. The traditional method group: before ligation, put a 0.2 ~ 0.3 cm 10 # polyethylene tubing under the line, and then ligate the LAD. After confirming that myocardial ischemia has been induced by ligation, close the chest cavity layer by layer by suturing the skin and muscle, and place two coils on both sides of the incision, leaving them outside the body. After 40 min of ischemia, the polyethylene tube was drawn out for perfusion for 24 h. The control group was treated by sham operation by receiving the same surgical interventions. For each group, five biological replicates were prepared. We used 0.12ml/10 g of 1% pentobarbital sodium for anesthesia, and after death from anesthesia, we bled the abdominal aorta to extract the heart tissue.

The slices of heart tissue were placed in TTC incubation solution (T8877-10 g, Sigma) preheated at 37 ℃ in advance, and incubate them in a 37 ℃ water bath in dark for 30 min. We then gently shaked the tissue at intervals of 10 min to evenly contact the staining solution, and observed the staining results.

### RNA extraction and sequencing

We used liquid nitrogen grounding method for myocardial tissue homogenization, and 10 to 50 mg of each sample was used to extract RNA. Total RNA was extracted using TRIZOL (Ambion). DNAs were removed by treating total RNA with RQ1 DNase (Promega). To detect the quality and count the number of the purified RNAs, the absorbance was measured at 260 nm/280nm (A260/A280) using smartspec plus (BioRad). Then, the integrity of total RNA was validated by 1.5% agarose gel electrophoresis.

Next, 1 µg total RNA was used to construct RNA-seq libraries for each sample. mRNAs were captured using oligo-d(T) capture Beads (VAHTS, Vazyme, N401). The purified RNA was used to prepare directional RNA-seq libraries by Stranded RNA-seq Kit for Illumina platforms (KK8544, KAPA). Polyadenylated mRNAs were subjected to purification and fragmentation. The cDNA synthesis was done by reverse transcription kit(R323-01, Vazyme, China) at 42˚C for 5 min, 37 ˚C for 15 min, 85 ˚C for 5 s that was performed on the thermocycler(T100, Bio-Rad, USA). After end repair and A tailing, the DNAs were subjected to ligation with Diluted Roche Adaptor (KK8726). After purifying the ligated products and fractioning the size to 300-500bps, the ligated products were subjected to amplification, purification, and quantification and were stored at -80℃ for sequencing. The second cDNA strand, which is marked with dUTP, is not amplified for sequencing specific strand.

For high-throughput sequencing, the libraries were constructed according to the manufacturer’s protocols and were sequenced with the Illumina NovaSeq 6000 platform for 150 nt paired-end sequencing.

### RNA-Seq raw data cleaning and alignment

Raw reads with no less than 2-N bases were first dropped. Then raw reads were trimmed using FASTX-Toolkit to remove low-quality bases and adaptors (Version 0.0.13). The short reads with no more than 16nt were also discarded. Subsequently, use HISAT2 [12] to align the clean reads to the GRCm39_M27 genome with 4 mismatches. And use uniquely mapped reads to count the reads number of genes and fragments per kilobase of transcript per million fragments mapped (FPKM) [13].

### Differentially expressed gene (DEG) analysis

DEGs were screened by using the R Bioconductor package DESeq2 [14] and identified by setting the corrected *p*-value < 0.05 and fold change > 2 or < 0.5 as the cut-off criteria.

### Alternative splicing analysis

ABLas pipeline was used to define and calculate the alternative splicing events (ASEs) and RASEs between the samples as described previously [15, 16]. In brief, nine types of ASEs were identified, including alternative 5’ splice site (A5SS), exon skipping (ES), alternative 3’splice site (A3SS), mutually exclusive 5’UTRs (5pMXE), mutually exclusive exons (MXE), cassette exon (exon inclusion), mutually exclusive 3’UTRs (3pMXE), A3SS&ES and A5SS&ES.

To evaluate the ASEs regulated by RBPs, Student’s *t*-test was performed to assess the significance of the ratio alteration of AS events. Those events which were significant at a *p*-value cutoff of 0.05 and a false discovery rate (FDR) of 5% were considered RASEs.

To construct the correlation network between RBPs and RASEs, we performed Pearson’s correlation analysis between the RASE ratios and the expression levels of differentially expressed RBPs (DE RBPs). The *p*-value < 0.01 and Pearson’s correlation coefficient > 0.95 were set as the criteria for DE RBP-RASE pairs.

### RT-qPCR experiment

For the validation of DEGs and RASEs, we performed reverse transcription and quantitative polymerase chain reaction (RT-qPCR) experiment. Specific primers were designed for each DEGs and RASEs and were provided in supplemental Table S1. GAPDH (glyceraldehyde-3-phosphate dehydrogenase) was used as a control gene for assessing the relative expression of specific genes. cDNA synthesis was done by standard procedures and RT-qPCR was performed on the Bio-Rad S1000 with Hieff™ qPCR SYBR® Green Master Mix (Low Rox Plus; YEASEN, China). The concentration of each transcript was then normalized to GAPDH mRNA level using 2^−ΔΔCT^ method [17]. Four biological replicates, and three technical replicates for each, were picked for the validation experiments.

### Protein-protein interaction analysis

For protein-protein interaction analysis, the symbols of selected genes were served as input to calculate and construct protein-protein interaction network through STRING database (https://cn.string-db.org/; Version: 12.0).

### Functional enrichment and statistical analysis

To sort out the DEGs’ functional groups, Kyoto Encyclopedia of Genes and Genomes (KEGG) pathways and Gene Ontology (GO) terms. were identified by utilizing KOBAS 2.0 server [18]. To define the enrichment of each term, Benjamini-Hochberg FDR controlling procedure and Hypergeometric test were used. As we have tested the that variances were equal and that the data were normally distributed for RT-qPCR results, we used two-tail unpaired Student’s *t*-test to obtain statistical significance. The statistical tests were performed with R software (v4.2.3).

## Results

### MIRI extensively regulated the global transcriptome profiles in the mouse model

Myocardial ischemia-reperfusion, clinical treatment for myocardial infarction, could induce myocardial injury and cardiomyocyte death, reducing its curative effects [19]. The underlying mechanisms of MIRI remain unclear. A MIRI mouse model was constructed using male C57BL/6 mice. The TTC staining result showed the obvious heart injury for myocardial tissues compared with the normal tissues (Figure S1A). Then five injured and adjacent normal myocardial tissues were extracted for RNA-seq experiments. After the qualified reads had been aligned to the mouse genome, one injured sample was removed due to its aberrant expression patterns compared with other ones. Principal component analysis (PCA) of all expressed genes indicated that there was a clear separation between the model (injured) and normal samples (Figure S1B). According to the DEG analysis, a total of 3265 DEGs were upregulated and 3459 downregulated between the model and normal samples (Figure S1C), indicating that extensive transcriptome alternations were induced by MIRI. Hierarchical clustering heatmap demonstrated the obvious and consistent expression patterns of DEGs (Figure S1D). We then performed functional enrichment analyses of these up- and down-regulated DEGs. The results demonstrated that up-regulated DEGs were mostly enriched in inflammatory and immune responses, cell cycle, and cell migration pathways (Figure S1E), while down-regulated DEGs in mitochondrial metabolism-associated pathways (Figure S1F), consistent with previous conclusions [19]. These results demonstrated that the global transcriptome profile was dramatically regulated by MIRI.

### The alternative splicing analyses of cardiac tissues in MIRI

**Fig. 1:**
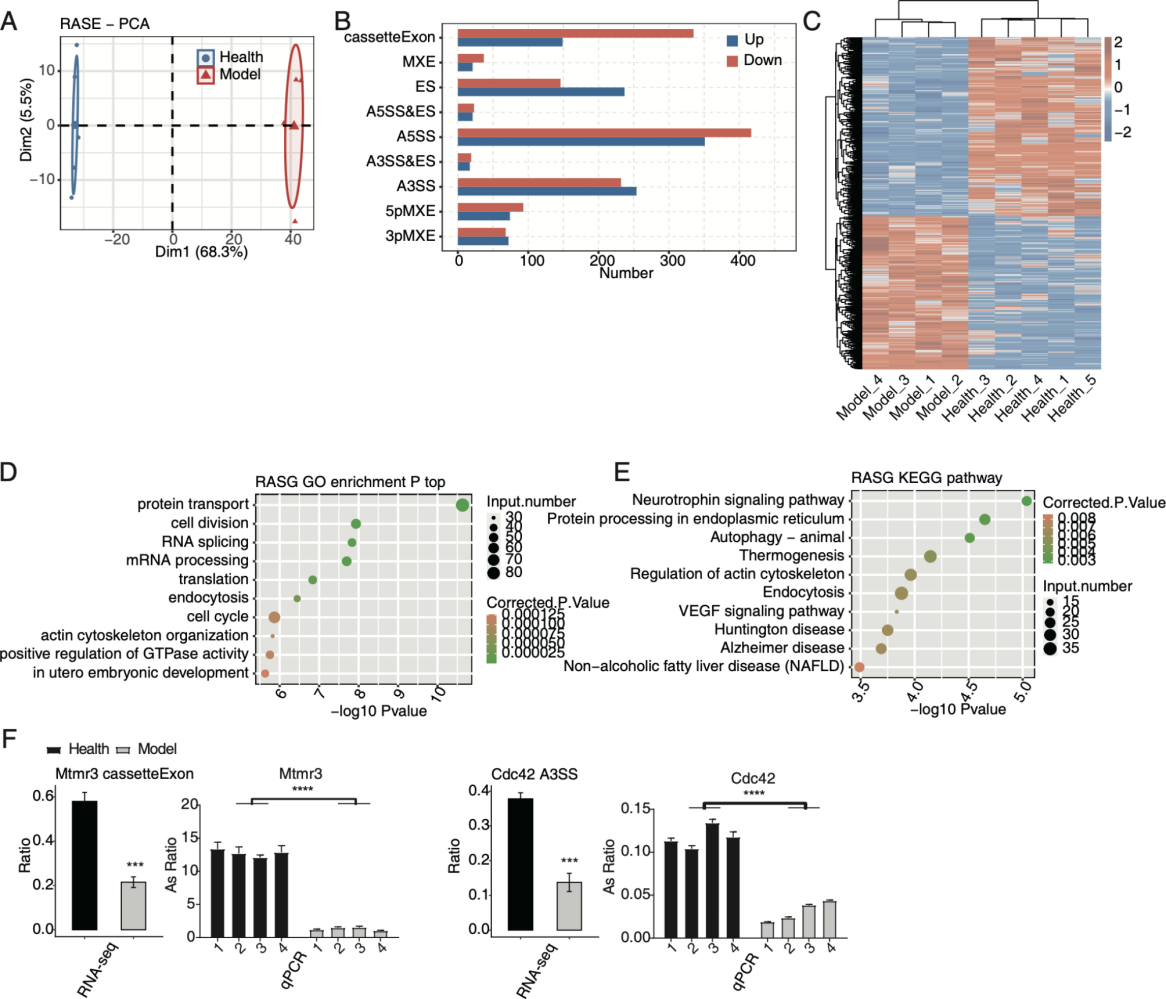
The AS analysis of cardiac tissues in MIRI. (A) PCA based on ratio values of all RASEs’ different expression levels. (B) Bar plot showing the RASEs. (C) Hierarchical clustering heatmap of the RASEs based on ratio values. (D) Scatter plot shows the top ten GO BP results of the RASGs. (E) Scatter plot shows the top ten KEGG pathways of the RASGs. (F) Bar plot shows the statistical differences and expression patterns of some important genes’ RASEs from RT-qPCR and RNA-seq validation. The Y-axis represent the AS ratio between model event and alternative event. Mean ± SEM is represented by error bars. Student’s *t*-test; *** *P*-value < 0.001, ** *P*-value < 0.01, * *P*-value < 0.05

A recent study has highlighted the influence of AS on cardiovascular diseases (CVDs) [20], but the underlying mechanisms of AS profiles being regulated in MIRI are largely unknown. Thus, we systematically explored the dysregulated AS profiles in MIRI mouse models. After aligning reads onto mouse genome, junction spliced reads were used as input for AS analysis. A toal of 2566 RASEs were detected between the model and healthy samples using ABLas program with the *p*-value < 0.05. PCA by calculating RASE ratios showed that the first component clearly separated the model and healthy samples, and that the first component could explain 68.3% of total variation (Fig. 1A). Then the RASEs were classified into nine types, with four types having more RASEs than others, including cassette exon, ES, A5SS, and A3SS (Fig. 1B). Another interesting finding was that the inclusion (up) or exclusion (down) number of cassette exon and ES events showed opposite trends (Fig. 1B), suggesting that MIRI model has the preference to exclude exons from primary transcripts. Then the clustering heatmap showing the ratios of all RASEs was plotted and high consistency was found among the replicates of each individual group, implying the high confidence of detected RASEs (Fig. 1C). We analyzed the enriched functions of RASGs, genes extracted from RASEs, using GO and KEGG databases, with protein transport, cell division/ cell cycle, RNA splicing, and endocytosis being the top enriched GO BP pathways (Fig. 1D). KEGG pathway analyses showed that autophagy-animal, protein processing in endoplasmic reticulum, neurotrophin signaling pathway, thermogenesis, and regulation of actin cytoskeleton were the top enriched pathways (Fig. 1E). These results together demonstrated genes involved in important pathways were regulated at the AS level in MIRI models. To validate the RASEs involved in pathways associated with MIRI, we designed specific PCR primers at splicing junctions and used RT-qPCR method to check the changed AS ratios of RASEs with high significance between the model and healthy samples. Both RNA-seq and RT-qPCR results indicated that events *Mtmr3* cassette exon and *Cdc42* A3SS were significantly changed between the model and healthy samples (Fig. 1F). In summary, MIRI model also greatly affected the AS profiles and perhaps substantially contributes to the progression of various diseases.

### Analysis of the differential expression of RBPs in MIRI

**Fig. 2:**
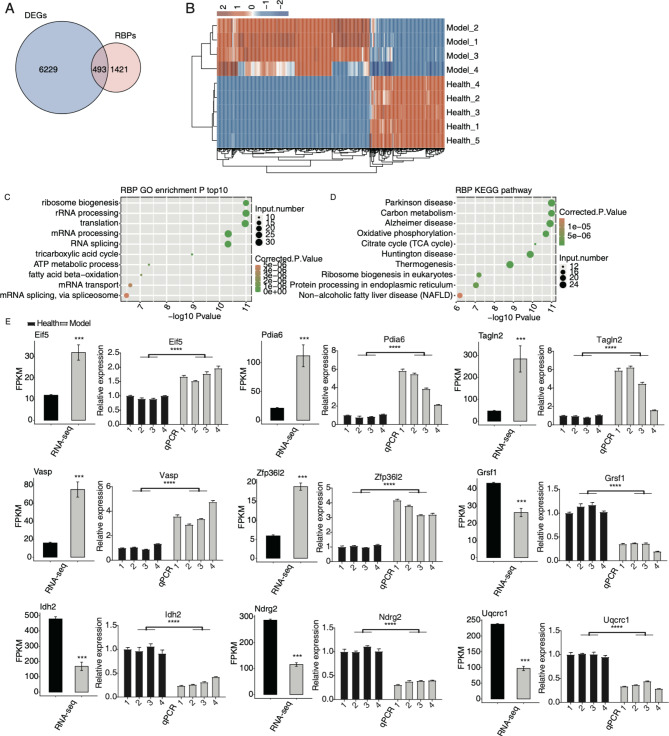
Analysis of the differential expression of RBPs in MIRI. (A) The overlapped genes between DEGs and RBPs are shown in venn diagram. (B) The expression levels of the DE RBPs are shown in hierarchical clustering heatmap. (C) The scatter plot shows the top ten GO BP results of the DE RBPs. (D) The scatter plot shows the top ten KEGG pathways of the DE RBPs. (E) Bar plot shows the statistical differences and expression patterns of the DEGs of some critical RBPs. Mean ± SEM is represented by error bars. Student’s *t*-test; *** *P*-value < 0.001; **** *P*-value < 0.0001

RBPs are associated with diseases and have essential roles in post-transcriptional regulation, including AS [21, 22]. We then analyzed the RBP expression in MIRI model to decipher the underlying molecular mechanisms of RASEs. The RBP gene list, containing 1914 genes, was obtained from published study [22]. First, 493 DE RBPs were identified by overlapping RBPs and DEGs (Fig. 2A). Hierarchical clustering heatmap also revealed the consistency in DE RBPs’ expression changes between the model and healthy samples (Fig. 2B). By checking their functions, we found DE RBPs were enriched in translation and mRNA processing pathways, as well as energy metabolism pathways (Fig. 2C). Interestingly, KEGG enrichment analysis demonstrated that they were highly enriched in several neurodegenerative diseases and carbon metabolism pathways (Fig. 2D), consistent with the previous summary of dysregulated RBPs in neurodegenerative diseases [23]. Protein-protein interaction (PPI) analysis indicated that several interactive networks were formed and may have potential regulatory functions, including the ribosome complex and ubiquinone oxidoreductase complex (Figure S2). To confirm the dysregulated RBPs in the MIRI model, RT-qPCR experiment was conducted to validate the changed RNA levels of nine randomly selected RBPs, including *Eif5*, *Pdia6*, *Tagln2*, *Vasp*, *Zfp36l2*, *Grsf1*, *Idh2*, *Ndrg2*, and *Uqcrc1*. With four models and four healthy samples as input, the results showed significant expression differences between models and healthy samples. The results of RNA-seq and RT-qPCR demonstrated consistency in the changed patterns (Fig. 2E). These results showed that RBPs were also dysregulated in MIRI models and may be essential for disease formation through regulating the fate of associated RNAs.

### Correlation network between DE RBPs and RASGs was associated with MIRI

**Fig. 3:**
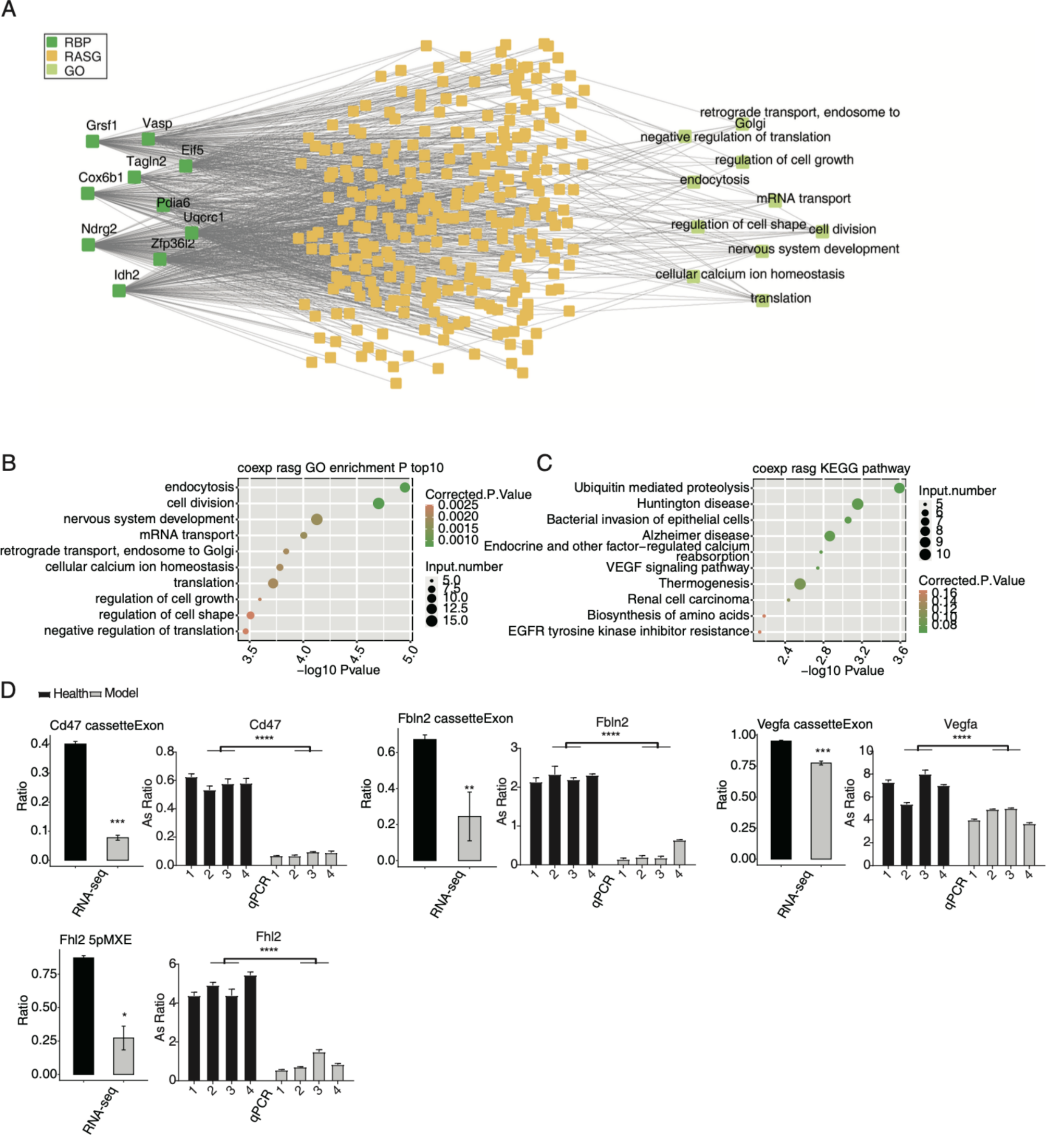
Correlation network between DE RBPs and RASGs associated with MIRI. (A) The network plot showing the RASG co-expressing with the ten RBPs. The enriched GO pathways for RASGs were shown in the right panel. (B) Scatter plot shows the top ten GO BP results of the co-expressed RASGs. (C) Scatter plot shows the top ten KEGG pathways of the co-expressed RASEs. (D) Bar plot shows the statistical differences and expression patterns of some important genes’ RASEs from RT-qPCR and RNA-seq validation. Mean ± SEM is represented by error bars. Student’s *t*-test; *** *P*-value < 0.001, ** *P*-value < 0.01, * *P*-value < 0.05

To further explore how dysregulated RBPs participate in the AS regulation in MIRI models, we constructed a correlation network between DE RBPs and RASGs, with DE RBP expression levels and RASE ratios being used as input data and Pearson’s correlation analysis performed to extract significant DE RBP-RASE pairs (|R| > 0.95 and *p*-value < 0.01). Finally, in this network, 848 RASEs correlated with 483 RBPs were obtained (Figure S3A). Analyses of the functions of the RASGs from the 848 RASEs indicated that they were associated with cell growth/division and important metabolism pathways (Figure S3A). Bubble plot also demonstrated that these RASGs were enriched in endocytosis, protein transport, and mitochondrial respiratory chain complex I assembly, the top three GO BP pathways (Figure S3B). Thermogenesis, Alzheimer disease, and bacterial invasion of epithelial cells were the top three KEGG pathways (Figure S3C). Dilated cardiomyopathy (DCM) pathway, including *Lama2*, *Tnnt2*, *Actg1*, *Atp2a2*, *Gnas*, *Tpm3*, *Itga6*, *Tpm1*, and *Pln*, was enriched in the KEGG pathway (Figure S3C). To further ensure the credibility of the result, we focused on the nine validated RBPs in Fig. 2E, and RBP Cox6b1, which was the most highly expressed and dysregulated RBP. Cox6b1 is an enzyme of the mitochondrial respiratory chain; a recent study demonstrated that metabolic enzymes have RNA binding activity and functions [24]. In total, 421 RASEs were correlated with these ten DE RBPs (Fig. 3A). Functional analyses of 290 RASGs from 421 RASEs revealed that they were closely associated with translation process, cell growth and division, and endocytosis (Fig. 3A). Then the top ten GO BP and KEGG pathways of the 290 RASGs were presented respectively, with endocytosis and cell division being the top two GO BP pathways (Fig. 3B), indicating the regulated cell cycle and cellular environment. Similarly, ubiquitin-mediated proteolysis was the top KEGG pathway (Fig. 3C). The vascular endothelial growth factor (VEGF) signaling pathway was also clustered in KEGG pathway (Fig. 3C). Finally, we selected several RASEs and validated the changed AS ratios by conducting RT-qPCR experiments. Cassette exon events of *Cd47, Fbln2, and Vegfa* were repressed in MIRI model; 5pMXE event of *Fhl2* was repressed in MIRI model (Fig. 3D). These results suggest that DE RBPs may participate in the AS regulation of *Cd47, Vegfa, Fhl2, Fbln2*, and other genes by bindig to RNA, thus promoting MIRI progression.

## Discussion

MIRI reduces the effect of myocardial reperfusion therapy for myocardial infraction, and its molecular pathogenesis should be deeply investigated. It has been shown that several factors, including oxidative stress, intracellular Ca^2+^ overload, physiological pH value, and inflammation, have been regarded as mediators of MIRI [19]. In this study, we constructed a MIRI mouse model, extracted injured and healthy myocardial tissues to explore the whole transcriptome alterations. In addition to the known dysregulated gene expression patterns, we also identified the AS profile changes between model and healthy samples. We found that the RASEs showed distinct features and their genes were enriched in several important pathways associated with MIRI. Finally, we constructed an RBP-RASE co-regulation network to identify the key modulators of these RASEs. Our study demonstrated that AS regulation deeply participated in the pathogenesis of MIRI, and emphasized the critical regulatory roles of RBPs.

Firstly, we validated the global transcriptome alternations by identifying thousands of DEGs which were identified as the cellular mediators of MIRI. The functions of DEGs were mainly immune/inflammation response, mitochondrial functions, and oxidation − reduction process [25, 26]. These results suggest the reliability of transcriptome data generated from MIRI model and healthy mice. In total, 493 DE RBPs were identified among the DEGs. It has been demonstrated that transcriptional and post-transcriptional processes regulated by RBPs are essential for the remodeling and regenerative responses to cardiovascular tissue damage [27]. We found that in MIRI model, these DE RBPs were significantly associated with RNA splicing and translation, as well as the energy metabolisms, including TCA cycle and ATP metabolic process, suggesting that DE RBPs participate in multiple biological aspects. We found that several RBPs were tightly associated with MIRI. EIF5A increased dramatically due to hypoxia/reoxygenation, and could induce cardiac myocyte apoptosis by acting as a ligand promoting apoptosis [28]. ZFP36L2 regulates MIRI by directly associating with lncRNA PVT1 and attenuates mitochondrial fusion and fission, revealing that ZFP36L2 may be a novel molecular target for MIRI treatment [29]. Previous study demonstrated that after IDH2 had been deacetylated through SIRT3, the MIRI was attenuated, suggesting that IDH2 could act as a potential molecular target for treating MIRI [30]. In addition, enriched in TCA cycle, these DE RBPs could precede the development of heart failure and atrial fibrillation [31]. TCA cycle is mainly completed mitochondria, and its dysfunction is the cause of many human diseases [32], implying that may RBPs have metabolic functions and their interaction with RNAs could regulate both enzyme’s catalytic activity and RNA fate [24]. Together, these results demonstrate that DE RBPs play a critical role in MIRI through various functional mechanisms.

In addition to the dysregulated RBPs, global AS profile was also significantly regulated in MIRI model. AS regulation has been recently reported in CVDs; circRNAs and products of irregular AS events were emphasized in the origination and development of MIRI [20]. In the current study, AS profile was found to show a certain changing pattern between model and healthy samples. In model samples, ES events dramatically increased, while the opposite events, cassette exon, decreased (Fig. 1B). Based on published results, ES or cassette exon events were dominant AS types in mammalian cells, and these events were precisely regulated by spliceosome complexes [33]. The result indicates that some key splicing factors may be dysregulated and alter the normal splicing patterns in myocardial cells of MIRI model. Consistent with this hypothesis, DE RBPs were found to be enriched in RNA splicing via spliceosome pathways (Fig. 2C), emphasizing the key regulatory functions of RBPs in splicing processes. More importantly, we found that the RASGs were closely associated with MIRI. Cell division/cycle pathways emerged in enriched pathways. A previous study demonstrated that in mouse heart, the overexpression of p57^Kip2^, a cell cycle inhibitor, could attenuate ischemia-reperfusion injury (IRI) [34], suggesting that the dysfunction of cell cycle RASGs may be a protective factor for MIRI. RASGs were enriched in autophagy and endocytosis pathways, which were together enhanced in the ischemia and reperfusion phase, suggesting their beneficial and detrimental effects during MIRI [35]. Among the RASGs, *Mtmr3* regulates the formation and size of autophagosome, and finally affects the autophagic activity [36]. The enriched autophagy and endocytosis pathways for RASGs indicate the important functions of AS regulation in MIRI and the following recovery. *Cdc42*, a cell division cycle regulatory gene, may play a role in myocardial I/R by affecting the cardiacmyocyte apoptosis rate through JNK, Bcl-2 and Bax signal pathway [37]. Irregular AS patterns of these two genes may affect the final protein functions. These results together demonstrate that AS patterns of genes have a profound impact on the progression of MIRI. Further studies are needed to investigate the functional mechanisms and functions of these dysregulated AS isoforms in MIRI.

Finally, we constructed the regulatory network between DE RBPs and RASEs. We found over 30% RASEs were correlated with DE RBPs, suggesting the regulatory roles of RBPs in AS profiles. Then nine validated DE RBPs were selected together with RASEs correlated with them, and several important RASGs were found to emerge, including *Cd47, Fbln2, Vegfa*, and *Fhl2*. CD47, a transmembrane protein, has been used to explore MIRI treatment by delivering anti-apoptotic miRNAs via forming complexes with extracellular vesicles (EVs) [38]. Meanwhile, acute CD47 inhibition could decrease cardiac damage and improve cardiac contractile function, suggesting that anti-CD47 could serve as a therapeutic strategy for treating ischemic heart [39]. Goltz et al. found that in Fhl2^−/−^ mouse, altered immune infiltration was found to respond to myocardial ischemia, contributing to smaller infarcts and better hemodynamic performance after MIRI [40]. Vascular endothelial growth factor A (VEGFA), could promote vascularization after acute myocardial infarction (AMI) by enhancing ROS generation and autophagy mediated by ER stress [41]. The splicing patterns of these genes may greatly change the encoded proteins and functions. There is a strong possibility that DE RBPs participate in important biological processes of MIRI through modulating the AS patterns of downstream genes. This regulatory process explains how RBPs are involved in myocardial diseases in the post-transcriptional level, which needs to be further validated. Meanwhile, the dysregulated expression of RBPs also needs to be validated by other methods in future.

## Conclusion

In summary, our study highlights the important roles of AS and RBPs in the pathogenesis of MIRI using mouse models. We propose that DE RBPs and their regulated AS genes participate in RNA splicing, metabolic processes, and immune/inflammatory response pathways to modulate MIRI progression. Several important RBPs, AS events and genes could serve as novel therapeutic targets in treating MIRI by designing accurate molecular medicine in the future. Our study extends the understanding of the molecular pathogenesis of MIRI and emphasizes the important roles of post-transcriptional regulation.

### Electronic supplementary material

Below is the link to the electronic supplementary material.

Supplementary Material 1

Supplementary Material 2

## Data Availability

The datasets generated during the current study are available in the EMBL repository with accession number PRJEB59007.

## Abbreviations

MIRI: myocardial ischemia-reperfusion injury
AS: alternative splicing
RBPs: RNA binding proteins
DEG: differentially expressed gene
RASGs: regulated alternative splicing genes
DE RBPs: differentially expressed RBPs
RASEs: regulated alternative splicing events
AMI: Acute myocardial infarction
CVDs: cardiovascular diseases
MI: myocardial infarction
RNP: ribonucleoprotein
LAD: anterior descending branch
ASEs: alternative splicing events
A5SS: alternative 5’ splice site
ES: exon skipping
A3SS: alternative 3’splice site
5pMXE: mutually exclusive 5’UTRs
MXE: mutually exclusive exons
3pMXE: cassette exon, mutually exclusive 3’UTRs
FDR: false discovery rate
KEGG: Kyoto Encyclopedia of Genes and Genomes
GO: Gene Ontology
PCA: Principal component analysis
BP: biological process
PPI: Protein-protein interaction
VEGF: vascular endothelial growth factor
IRI: ischemia-reperfusion injury
EVs: extracellular vesicles
VEGFA: Vascular endothelial growth factor A

## References

[1] Pollard, Timothy J (2000). The acute Myocardial Infarction. Prim Care Clin Office Pract.

[2] Buja LM (2005). Myocardial ischemia and reperfusion injury. Cardiovasc Pathol.

[3] Ge H, Lin W, Lou Z, Chen R, Shi H, Zhao Q, Lin Z (2022). Catalpol alleviates myocardial ischemia reperfusion injury by activating the Nrf2/HO-1 signaling pathway. Microvasc Res.

[4] Vos PD, Leedman PJ, Filipovska A, Rackham O. Modulation of miRNA function by natural and synthetic RNA-binding proteins in cancer. *Cellular and Molecular Life Sciences CMLS* 2019, 76(303). 10.1007/s00018-019-03163-9 PMC11105495 31165201

[5] Guo W, Shi X, Liu A, Yang G, Yu F, Zheng Q, Wang Z, Allen DG, Lu Z (2011). RNA binding protein QKI inhibits the ischemia/reperfusion-induced apoptosis in neonatal cardiomyocytes. Cell Physiol Biochem.

[6] Ding HS, Yang J, Chen P, Bo SQ, Ding JW, Yu QQ (2013). The HMGB1-TLR4 axis contributes to myocardial ischemia/reperfusion injury via regulation of cardiomyocyte apoptosis. Gene: An International Journal Focusing on Gene Cloning and Gene Structure and Function.

[7] Baeza-Centurion P, Miñana B, Schmiedel JM, Valcárcel J, Lehner B. Combinatorial Genetics reveals a Scaling Law for the effects of mutations on splicing. Cell; 2019. 10.1016/j.cell.2018.12.010 30661752

[8] Yang X, Coulombe-Huntington J, Kang S, Sheynkman GM, Vidal M (2016). Widespread expansion of Protein Interaction capabilities by Alternative Splicing. Cell.

[9] Baralle FE, Giudice J. Alternative splicing as a regulator of development and tissue identity. Nat Rev Mol Cell Biol 2017, 18(7). 10.1038/nrm.2017.27 PMC6839889 28488700

[10] Bell JR, Raaijmakers A, Curl CL, Reichelt ME, Harding TW, Bei A, Ng D, Erickson JR, Petroff MV, Harrap SB (2015). Cardiac CaMKIIδ splice variants exhibit target signaling specificity and confer sex-selective arrhythmogenic actions in the ischemic-reperfused heart. Int J Cardiol.

[11] Khan MM, Gandhi C, Chauhan N, Stevens JW, Motto DG, Lentz SR, Chauhan AK (2012). Alternatively-spliced Extra Domain a of Fibronectin promotes Acute inflammation and brain Injury after cerebral ischemia in mice. Stroke.

[12] Kim D, Langmead B, Salzberg SL (2015). HISAT: a fast spliced aligner with low memory requirements. Nat Methods.

[13] Trapnell C, Williams BA, Pertea G, Mortazavi A, Kwan G, Van Baren MJ, Salzberg SL, Wold BJ, Pachter L (2010). Transcript assembly and quantification by RNA-Seq reveals unannotated transcripts and isoform switching during cell differentiation. Nat Biotechnol.

[14] Love MI, Huber W, Anders S (2014). Moderated estimation of Fold change and dispersion for RNA-seq data with DESeq2. Genome Biol.

[15] Jin L, Li G, Yu D, Huang W, Cheng C, Liao S, Wu Q, Zhang Y (2017). Transcriptome analysis reveals the complexity of alternative splicing regulation in the fungus verticillium dahliae. BMC Genomics.

[16] Xia H, Chen D, Wu Q, Wu G, Zhou Y, Zhang Y, Zhang L (2017). CELF1 preferentially binds to exon-intron boundary and regulates alternative splicing in HeLa cells. Biochim et Biophys Acta (BBA)-Gene Regul Mech.

[17] Livak KJ, Schmittgen TD (2001). Analysis of relative gene expression data using real-time quantitative PCR and the 2(-Delta Delta C(T)) method. Methods.

[18] Xie C, Mao X, Huang J, Ding Y, Wu J, Dong S, Kong L, Gao G, Li C-Y, Wei L (2011). KOBAS 2.0: a web server for annotation and identification of enriched pathways and Diseases. Nucleic Acids Res.

[19] Hausenloy DJ, Yellon DM (2013). Myocardial ischemia-reperfusion injury: a neglected therapeutic target. J Clin Invest.

[20] Hasimbegovic E, Schweiger V, Kastner N, Spannbauer A, Traxler D, Lukovic D, Gyongyosi M, Mester-Tonczar J. Alternative splicing in Cardiovascular Disease-A Survey of recent findings. Genes (Basel) 2021, 12(9). 10.3390/genes12091457 PMC8469243 34573439

[21] Gebauer F, Schwarzl T, Valcarcel J, Hentze MW (2021). RNA-binding proteins in human genetic Disease. Nat Rev Genet.

[22] Hentze MW, Castello A, Schwarzl T, Preiss T (2018). A brave new world of RNA-binding proteins. Nat Rev Mol Cell Biol.

[23] Maziuk B, Ballance HI, Wolozin B (2017). Dysregulation of RNA binding protein aggregation in neurodegenerative disorders. Front Mol Neurosci.

[24] Curtis NJ, Jeffery CJ (2021). The expanding world of metabolic enzymes moonlighting as RNA binding proteins. Biochem Soc Trans.

[25] Gonzalez-Montero J, Brito R, Gajardo AI, Rodrigo R (2018). Myocardial reperfusion injury and oxidative stress: therapeutic opportunities. World J Cardiol.

[26] Wang J, Toan S, Zhou H (2020). New insights into the role of mitochondria in cardiac microvascular ischemia/reperfusion injury. Angiogenesis.

[27] de Bruin RG, Rabelink TJ, van Zonneveld AJ, van der Veer EP (2017). Emerging roles for RNA-binding proteins as effectors and regulators of Cardiovascular Disease. Eur Heart J.

[28] Seko Y, Fujimura T, Yao T, Taka H, Mineki R, Okumura K, Murayama K (2015). Secreted tyrosine sulfated-eIF5A mediates oxidative stress-induced apoptosis. Sci Rep.

[29] Wu F, Huang W, Tan Q, Guo Y, Cao Y, Shang J, Ping F, Wang W, Li Y (2021). ZFP36L2 regulates myocardial ischemia/reperfusion injury and attenuates mitochondrial fusion and fission by LncRNA PVT1. Cell Death Dis.

[30] Ma LL, Kong FJ, Ma YJ, Guo JJ, Wang SJ, Dong Z, Sun AJ, Zou YZ, Ge JB (2021). Hypertrophic preconditioning attenuates post-myocardial infarction injury through deacetylation of isocitrate dehydrogenase 2. Acta Pharmacol Sin.

[31] Bullo M, Papandreou C, Garcia-Gavilan J, Ruiz-Canela M, Li J, Guasch-Ferre M, Toledo E, Clish C, Corella D, Estruch R (2021). Tricarboxylic acid cycle related-metabolites and risk of atrial fibrillation and Heart Failure. Metabolism.

[32] Briere JJ, Favier J, Gimenez-Roqueplo AP, Rustin P (2006). Tricarboxylic acid cycle dysfunction as a cause of human Diseases and Tumor formation. Am J Physiol Cell Physiol.

[33] Ule J, Blencowe BJ (2019). Alternative Splicing Regulatory networks: functions, mechanisms, and evolution. Mol Cell.

[34] Haley SA, Zhao T, Zou L, Klysik JE, Padbury JF, Kochilas LK (2008). Forced expression of the cell cycle inhibitor p57Kip2 in cardiomyocytes attenuates ischemia-reperfusion injury in the mouse heart. BMC Physiol.

[35] Ma S, Wang Y, Chen Y, Cao F (2015). The role of the autophagy in myocardial ischemia/reperfusion injury. Biochim Biophys Acta.

[36] Taguchi-Atarashi N, Hamasaki M, Matsunaga K, Omori H, Ktistakis NT, Yoshimori T, Noda T (2010). Modulation of local PtdIns3P levels by the PI phosphatase MTMR3 regulates constitutive autophagy. Traffic.

[37] Xu X, Kong L, Song X, Hao Z, Yuan F (2017). Effect of Cdc42 on myocardial ischemia-reperfusion of rats. Cell Mol Biol (Noisy-le-grand).

[38] Wei Z, Chen Z, Zhao Y, Fan F, Xiong W, Song S, Yin Y, Hu J, Yang K, Yang L (2021). Mononuclear phagocyte system blockade using extracellular vesicles modified with CD47 on membrane surface for Myocardial Infarction reperfusion injury treatment. Biomaterials.

[39] Zhang S, Yeap XY, DeBerge M, Naresh NK, Wang K, Jiang Z, Wilcox JE, White SM, Morrow JP, Burridge PW (2017). Acute CD47 Blockade during ischemic myocardial reperfusion enhances phagocytosis-Associated Cardiac Repair. JACC Basic Transl Sci.

[40] Goltz D, Hittetiya K, Gevensleben H, Kirfel J, Diehl L, Meyer R, Buttner R (2016). Loss of the LIM-only protein Fhl2 impairs inflammatory reaction and scar formation after cardiac ischemia leading to better hemodynamic performance. Life Sci.

[41] Zou J, Fei Q, Xiao H, Wang H, Liu K, Liu M, Zhang H, Xiao X, Wang K, Wang N (2019). VEGF-A promotes angiogenesis after acute Myocardial Infarction through increasing ROS production and enhancing ER stress-mediated autophagy. J Cell Physiol.

