# Supplementary material for: Genome-wide analysis revealed the dysregulation of RNA binding protein-correlated alternative splicing events in myocardial ischemia reperfusion injury
Source: BMC Med Genomics. 2023 Oct 19;16:251. doi: 10.1186/s12920-023-01706-5 (PMC10585833; doi:10.1186/s12920-023-01706-5)
Supplement: Supplementary file 1 — Supplementary Material 1 [file 12920_2023_1706_MOESM1_ESM.pdf]

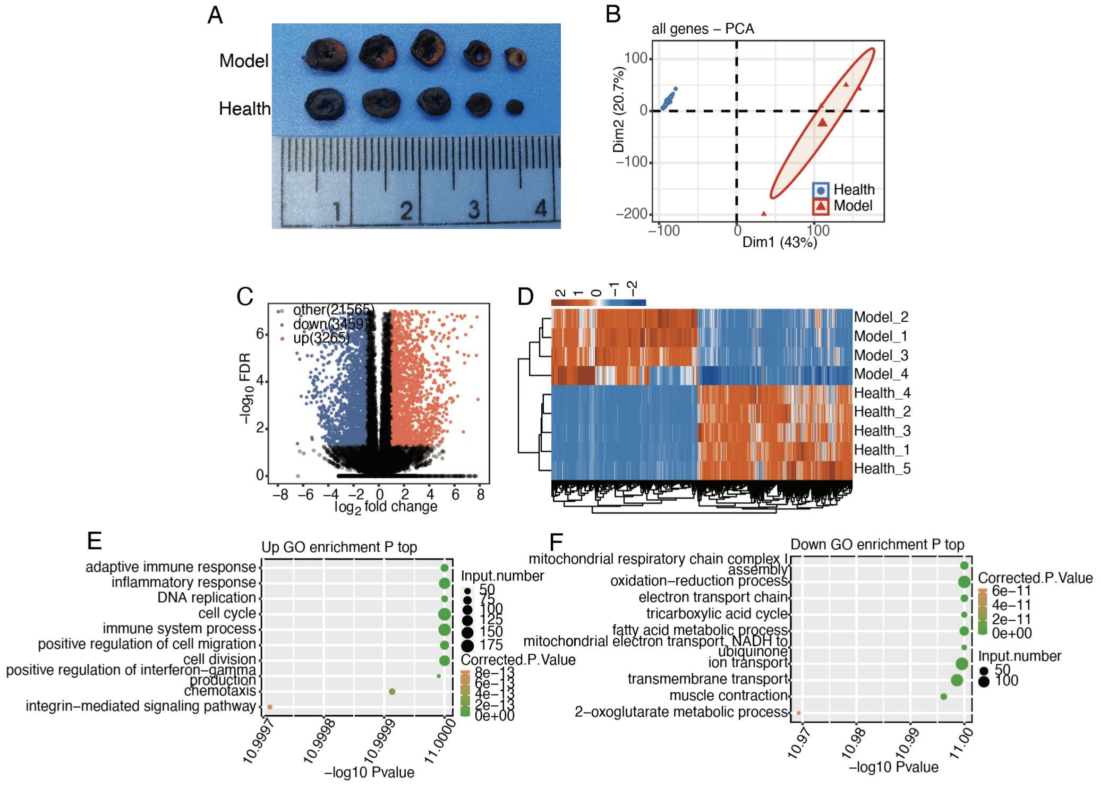

Fig S1. Transcriptome analysis of differentially expressed genes of cardiac tissues in myocardial ischemia reperfusion injury.

(A) The TTC staining result for the model and health heart tissues.

(B) PCA base on FPKM value of all detected genes. The ellipse for each group is the confidence ellipse.

(C) Volcano plot showing all differentially expressed genes (DEGs) between Model and Health

samples.

(D) Hierarchical clustering heatmap showing the expression pattern of all DEGs.

(E) The Scatter plot exhibiting the most enriched GO biological process results of the upregulated DEGs.

(F) The Scatter plot exhibiting the most enriched GO biological process results of the downregulated DEGs

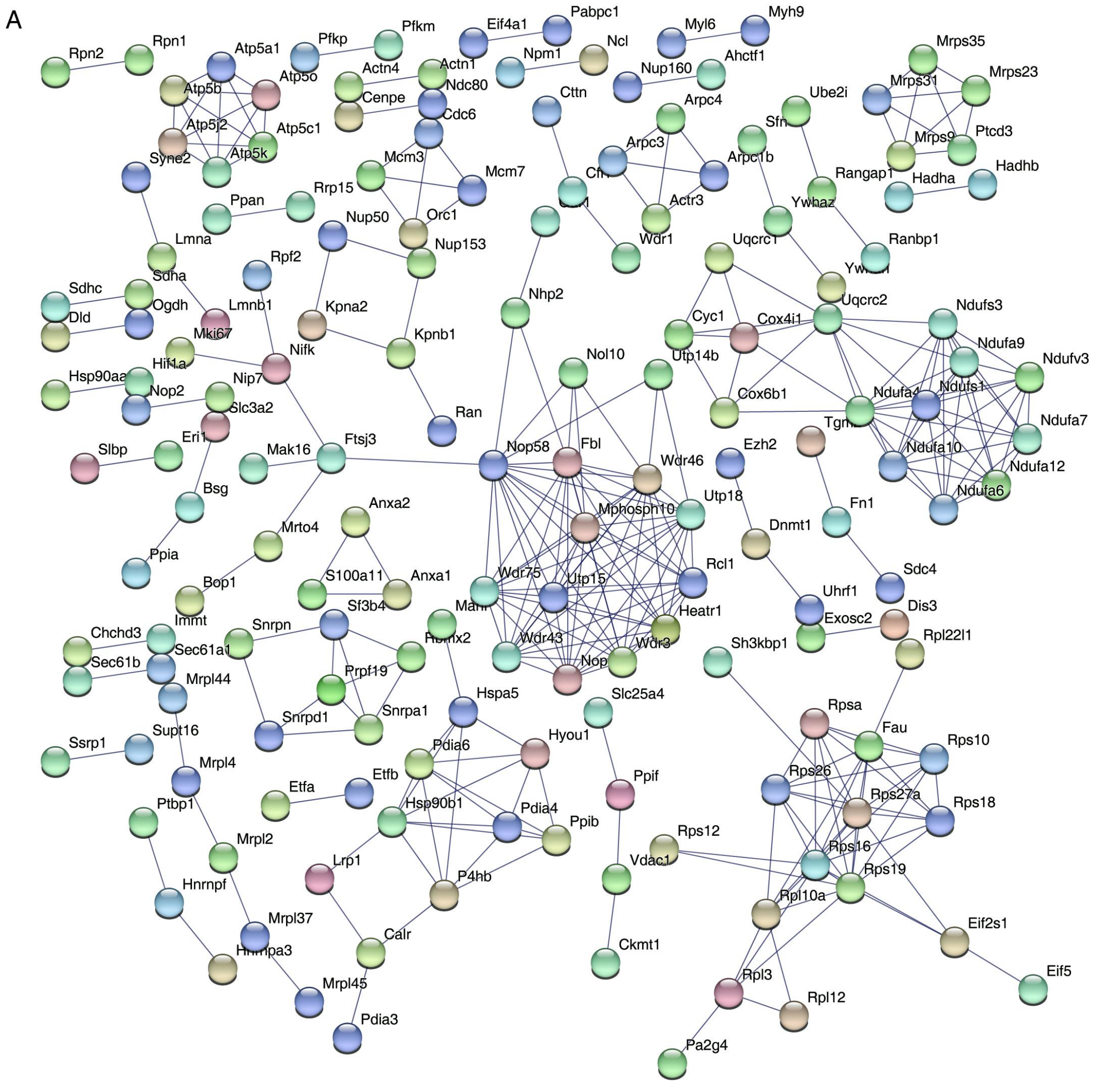

Fig S2. Protein-protein interaction mapping of the 493 RBPs.

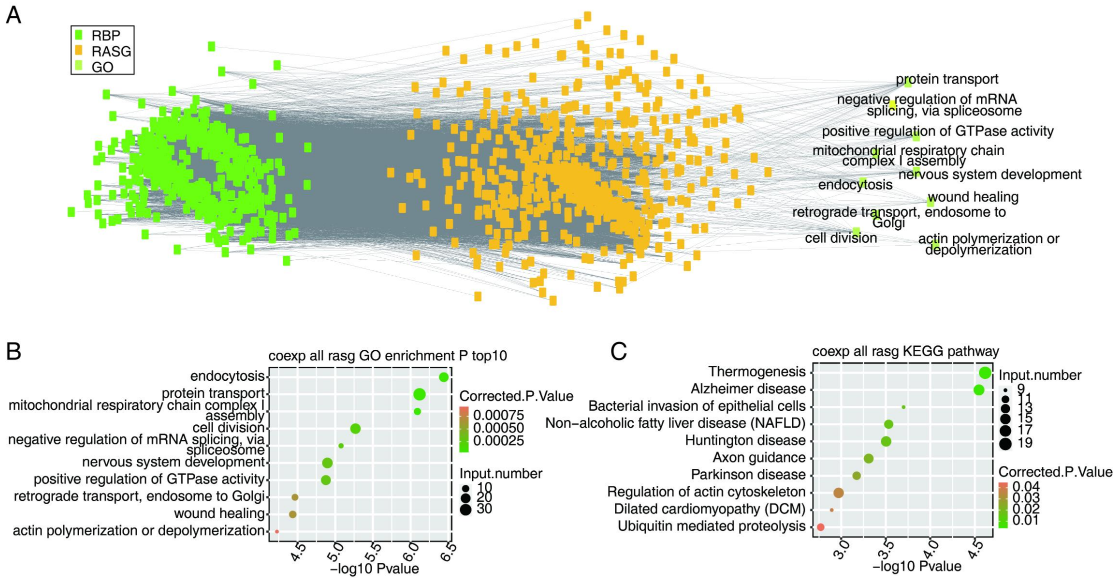

Fig S3. Co-expressing analysis of all RBPs and regulated alternative splicing events.

(A) The network plot showing all RBPs co-expressing RASG. The enriched GO pathways for RASGs were shown in the right panel.

(B) Scatter plot exhibiting the most enriched GO biological process results of the co-expressed RASGs.

(C) Scatter plot exhibiting the most enriched KEGG pathway results of the co-expressed RASEs.
