# Supplementary material for: Genome-wide analysis revealed the dysregulation of RNA binding protein-correlated alternative splicing events in myocardial ischemia reperfusion injury
Source: BMC Med Genomics. 2023 Oct 19;16:251. doi: 10.1186/s12920-023-01706-5 (PMC10585833; doi:10.1186/s12920-023-01706-5)
Supplement: Supplementary file 2 — Supplementary Material 2 [file 12920_2023_1706_MOESM2_ESM.pdf]

Table S1. The PCR primer sequence table. F: forward; R: reverse; M: model; AS: alternative spliced.

Tagln2-F	GAAAGAACATGGCTTGTGT
Tagln2-R	TAGGAAACCAGTTGGGATC
Grsf1-F	GCTATTAATGTCCTTACCAGTC
Grsf1-R	AAGTCTAGGCAACCTTCAC
Vasp-F	AGAACAAGAGAAGGTGAGAC
Vasp-R	CTGGCTGCTGATTGGATA
Idh2-F	CTTGAGCCAGGATGTCAG
Idh2-R	GCACTATAAGACTGACTTCG
Uqcrc1-F	TTCCAGACCTTCAACATCTC
Uqcrc1-R	CTTGCAGGAAGAAGACCAT
Pdia6-F	AGGATTCCCTACAATCAAGA
Pdia6-R	GGGCATTATCAGAGAACAAA
Eif5-F	ACACTTAGGAAGGTCTTGG
Eif5-R	TCAATAGAGCAACGAAGGA
Ndrg2-F	AACATACAAGCCTTCTCAAC
Ndrg2-R	GCCAGAGTAAGCAAGGAA
Zfp36l2-F	GCATATTCATGTACCACCAA
Zfp36l2-R	CCGAACTTGCCTTAAATCTT
Fhl2-M/AS-F	TCACAGGTATTGGCATAGAGT
Fhl2-M-R	CAGCATCAGGATTGCTGAAA
Fhl2-AS-R	CAAGACCAAGATTGCTGAAA
Cd47-M/AS-F	CCCTTTTGATTTCAGGTTTG
Cd47-AS-R	TCTCTCCACTCGACAAACTT
Cd47-M-R	TGGTTGGAAGCGACAAACTT
Vegfa-M/AS-F	GACGATGATGGCGTGGTGGT
Vegfa-AS-R	GTACTTGCAGATGTGACAAG
Vegfa-M-R	AGCCAGAAAAATGTGACAAG
Fbln2-M/AS-F	GTGTCGTGTTGTTGGGGACA
Fbln2-AS-R	TCGTCTTGGTCTTCACAGGA
Fbln2-M-R	TCATTGATGTCTTCACAGGA
Mtmr3-M/AS-F	TTTGAAGTGGCAGCAATAGG
Mtmr3-AS-R	ATCAAACGTGGAATTGTGGG
Mtmr3-M-R	ACCACTGCAGGAATTGTGGG
Cdc42-M-F	TTAGGCCTTTCTGTGTGAGG
Cdc42-AS-F	TCAGACCTCTCTGTGTGAGG
Cdc42-M/AS-R	CCTTTCTTGCTTGTTGGGAC
Gapdh-F	GGAGATGCTCAGTGTTGG
Gapdh-R	TGACAATGAATACGGCTACA
